# Nitrogen Substrate Impacts *Microcystis aeruginosa* Exometabolome Composition

**DOI:** 10.1111/1758-2229.70189

**Published:** 2025-09-02

**Authors:** Caroline M. Peck, Lauren N. Hart, Roland Kersten, Jenan J. Kharbush

**Affiliations:** ^1^ Department of Earth and Environmental Sciences University of Michigan Ann Arbor Michigan USA; ^2^ Program in Chemical Biology University of Michigan Ann Arbor Michigan USA; ^3^ Life Sciences Institute University of Michigan Ann Arbor Michigan USA; ^4^ Department of Medicinal Chemistry University of Michigan Ann Arbor Michigan USA

**Keywords:** exuded metabolites, harmful algal blooms, nontargeted metabolomics, toxic cyanobacteria

## Abstract

*Microcystis aeruginosa* is a toxic cyanobacteria species that is often abundant during cyanobacterial harmful algal blooms (cyanoHABs) in freshwaters. This study examined how growth on different nitrogen substrates influences the exometabolome of toxic and non‐toxic strains of *M. aeruginosa.* We used untargeted metabolomics, with liquid chromatography‐mass spectrometry of metabolites followed by feature‐based molecular networking and in silico metabolite annotation. Molecules released by *M. aeruginosa* varied based on the type of N substrate provided: the exometabolomes of cultures grown on ammonium and urea were more similar to each other and distinct from those grown on nitrate, suggesting that different assimilatory energetic requirements between reduced and oxidised N substrates are an important driver of exometabolome composition. Amino acids and peptides were the dominant compound class among metabolites that were significantly different between N treatments, but responses to N substrate were also reflected in altered extracellular concentrations of lipids, cyanotoxins, and photoprotectants. These differences in the molecular‐level response to the type of N substrate supplied support that environmental factors like changing N availability and oxidative stress may synergistically influence *M. aeruginosa* strain fitness and community succession, as well as interactions between *M. aeruginosa* strains and other bacteria or cyanobacteria in the bloom community.

## Graphical abstract

We used untargeted metabolomics to identify differences in the compounds exuded into culture media by three strains of *M. aeruginosa* grown on three N substrate treatments (NO_3_^−^, NH_4_^+^, and urea). Results showed that the usage of different N substrates changes the exometabolome of toxic and non‐toxic strains of *M. aeruginosa.*

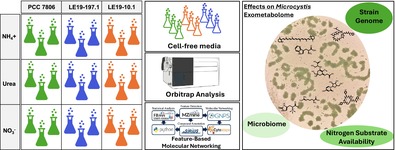

## 1 Introduction

The economic and health hazards posed by cyanobacterial harmful algal blooms (cyanoHABs) worldwide motivate ongoing efforts to characterise the factors that influence bloom biomass and toxicity, especially in freshwater systems. Lake Erie (North America) is a prime example of a critical natural resource threatened by cyanoHABs, supplying drinking water for 11 million people in both the United States and Canada, and supporting billions of dollars in fishing and tourism. Since the late 1990s, the western basin of Lake Erie has experienced annual summer cyanoHABs driven by excess phosphorus (P) and nitrogen (N) loading from agricultural runoff delivered primarily via the Detroit, Maumee, and Sandusky rivers (Allinger and Reavie 2013). These blooms are often dominated by the toxic cyanobacteria *Microcystis aeruginosa* . The most ubiquitous cyanotoxins produced by *M. aeruginosa* are microcystins, a class of hepatotoxins that can cause vomiting, diarrhoea, headaches, and skin blistering when ingested (reviewed in Bouaïcha et al. 2019). In multiple instances, city governments have issued water advisories when overgrowth or lysing of *M. aeruginosa* has resulted in unsafe levels of cyanotoxins in Lake Erie, such as during the 2014 shutdown of the water supply for the entire city of Toledo (Steffen et al. 2017).

N availability is critical to the ecology of blooms dominated by non‐N_2_‐fixing cyanobacteria like *M. aeruginosa*, and for the production of the N‐rich microcystin toxins (Gobler et al. 2016, Horst et al. 2014). Because *M. aeruginosa* cannot fix N_2_ gas, they must use oxidised forms such as nitrate (NO_3_^−^) or reduced forms including ammonium (NH_4_^+^) and dissolved organic N (DON), a complex mixture of organic molecules that includes substrates like urea, amino acids, and peptides. Generally, NH_4_^+^ is considered the most bioavailable form of N for phytoplankton, because NH_4_^+^ requires less energy to assimilate into cellular biomass, while the assimilation of other forms like NO_3_^−^ or DON requires more energy and cellular machinery (Flores and Herrero 2005; Veaudor et al. 2019).

The dominant form of bioavailable N and relative composition of the total N pool changes throughout the cyanoHAB season from July to September (Kharbush et al. 2023). The pattern is relatively consistent between years: NO_3_^−^ is the dominant form in early summer during bloom initiation but is consumed as the bloom progresses, leaving DON as the most abundant form by late summer/early fall. NH_4_^+^ concentrations are consistently low (Kharbush et al. 2023; Boegehold et al. 2023), but NH_4_^+^ is likely regenerated via remineralisation of organic N by associated heterotrophic bacteria (Yan et al. 2023; Hoffman et al. 2022) and then rapidly taken up. These associated heterotrophs grow within and around colonies in the phycosphere, the region enriched in organic substrates exuded by phytoplankton cells (Seymour et al. 2017).

Genomic analysis suggests that phycosphere‐associated heterotrophs perform nutrient recycling functions for *M. aeruginosa* , and in turn rely on compounds excreted by the cyanobacteria (Smith et al. 2021). They may also have impacts on the morphology and physiology of a colony; axenic *M. aeruginosa* has been shown to grow as single cells, rather than in colonies (Shen et al. 2011). *M. aeruginosa* strains in natural cyanoHAB communities have distinct microbiomes that differ spatially and temporally (Smith et al. 2021), but the underlying mechanisms driving these differences are unresolved.

One hypothesis is that microbiome species composition may be influenced by the metabolic capabilities of *M. aeruginosa* strains, including production and exchange of metabolites (Smith et al. 2021). Release of exometabolites into the environment is hypothesized as a major way that phytoplankton influence their microbiome composition (e.g., Briand et al. 2023), but characterisation of phytoplankton exometabolites and their ecological roles remains limited. Work in other environments showed that the composition of phytoplankton‐released compounds (the exometabolome) was different between algal species (Brisson et al. 2021), and that the released metabolites were preferentially incorporated by distinct groups of heterotrophs (Kieft et al. 2021, Mayali et al. 2023).

Understanding the role of phycosphere communities in supporting blooms of *M. aeruginosa* is also important for toxin production. Microcystin is synthesised via a large multi‐enzyme complex made up of a combination of non‐ribosomal peptide and polyketide synthases, encoded by the *mcy* biosynthetic gene cluster (Tillett et al. 2000). Toxigenic strains contain the full 10‐gene cluster of *mcy* genes, while non‐toxigenic strains typically lack *mcy* genes. Recently, a truncated, partial *mcy* genotype was also detected that appears to be ecologically successful as it was found across multiple bloom seasons in Lake Erie, and in a few instances, was the most abundant *mcy* genotype observed (Yancey et al. 2022). The product of this partial *mcy* genotype is likely a tetrapeptide with some inflammatory or hepatotoxic activity like microcystin (Yancey et al. 2024).

Community shifts in *M. aeruginosa* strain composition, from mostly toxigenic to non‐toxigenic strains, are often observed midway through the bloom as the overall amount of N decreases and the primary available N substrates change from inorganic to organic forms (Kharbush et al. 2023; Beversdorf et al. 2015). Toxin concentrations follow this community shift and are often higher in the early stages of the bloom and decrease as the bloom progresses, despite the persistence of high amounts of cyanobacterial biomass (Gobler et al. 2016; Berry et al. 2017).

This community succession could be related to N availability; the large amount of enzyme machinery combined with the N‐rich nature of microcystin (10 atoms of N per molecule) is consistent with prior work showing that toxigenic strains require more N than non‐toxigenic strains (Vézie et al. 2002, Downing et al. 2005, Wagner et al. 2021). *M. aeruginosa* strains contain similar genetic capabilities for N assimilation (Dick et al. 2021), but differences in N substrate usage could arise from differences in N metabolism and gene expression across strains, including toxin production capability (Pan et al. 2019; Alexova et al. 2011). N utilisation could also differ due to interactions with microbial members of the phycosphere, which may have capabilities to use and regenerate organic N and P that *M. aeruginosa* would otherwise be unable to access. These different strategies for N use and recycling could influence the competitiveness of different *M. aeruginosa* strains with changes in bioavailable N concentration and form during the bloom season, and help to explain observed community succession and toxin production patterns.

Oxidative stress could also be critical to understanding the ecology of toxigenic vs. non‐toxigenic strains. Under conditions of high oxidative stress, microcystins bind to proteins and protect them from damage from reactive oxygen species like H_2_O_2_ (Zilliges et al. 2011; Dziallas and Grossart 2011). The highest concentrations of H_2_O_2_ in WLE often precede the most toxic phase of the bloom (Cory et al. 2016), leading to the hypothesis that under these conditions microcystin‐producing strains may have a selective advantage over non‐toxigenic strains, which use other oxidative stress management strategies. Because of the high N requirement for microcystin biosynthesis, however, this advantage may disappear as inorganic N concentrations decrease. The community succession from toxic to non‐toxic strains may therefore be strongly related to strain‐level physiological differences or trade‐offs in oxidative stress protection and requirements for N, as proposed in a recent modelling study (Hellweger et al. 2022). Phycosphere interactions also play a key role in H_2_O_2_ detoxification (Smith et al. 2022).

Overall, the extent to which *M. aeruginosa* biology, bloom progression, and toxin production may be influenced by interactions with phycosphere communities is poorly understood. One reason is that we understand very little about the molecular composition of the exometabolome and what controls that composition. Given the links between N availability, toxin production, and *Microcystis* strain succession, this study examined how usage of different N substrates influences the exometabolome of toxic and non‐toxic strains of *M. aeruginosa*. We used untargeted metabolomics to identify differences in the compounds exuded into culture media by one axenic and two xenic strains of *M. aeruginosa*. Each strain was grown on three N substrate treatments: NO_3_^−^, NH_4_^+^, and urea. We hypothesised that assimilation of different N substrates would change the composition of exometabolites produced in different strains of *M. aeruginosa*, perhaps because of differences in assimilatory energetic requirements between N substrates. This study advances our knowledge on how strains of *M. aeruginosa* respond to N substrate at the molecular level, with implications for understanding their interactions with heterotrophs and other species of phytoplankton, as well as for cyanoHAB progression.

## 2 Methods

### 2.1 Strain Selection

Three strains were selected from the Western Lake Erie Culture Collection (WLECC, Yancey, Kiledal, et al. 2023) and the Catalogue of Microorganisms of the Biological Research Center of Institut Pasteur (PCC). Selected strains were LE19‐10.1 and LE19‐197.1 (WLECC) and PCC 7806 (Table 1), based on their ability to thrive in all experimental media conditions, their varying ability to produce microcystin (*mcy* present, absent, or partial genotypes), and the presence or absence of heterotrophic bacteria.

**TABLE 1:** Summary of strain characteristics.

Strain	Location	Date	*mcy*	Associated community
PCC 7806	Netherlands	January 1972	Present	Axenic
LE19‐10.1	Western Lake Erie	July 2019	Partial	Heterotrophs, *mLr* complete
LE19‐197.1	Western Lake Erie	August 2019	Absent	Heterotrophs, *mLr* incomplete

*Note: Mcy*: Indicates genomic presence of the operon that encodes the production of microcystin. The Associated Community column indicates other microbes within the culture, where the culture is either axenic or has heterotrophic bacteria present (Yancey, Kiledal, et al. 2023). Associated bacteria for LE19‐10.1 (GCA_027530565.1) are *Paracoccaceae* bacterium (GCA_027530395.1), *Limnobacter* sp. (GCA_027530465.1), *Cytophagales* bacterium (GCA_027530605.1), and *Novosphingobium* sp. (GCA_027530385.1), and for LE19‐197.1 are *Polaromonas* bacterium and *Novosphingobium* sp. The presence of the microcystin degradation pathway is indicated by “*mLr”* (Bourne et al. 2001; Dziga et al. 2013).

### 2.2 Culturing and Experimental Design

*M. aeruginosa* was cultured following the Western Lake Erie Culture Collection's liquid transfer protocol (https://sites.lsa.umich.edu/wleculturecollection/). Cultures were grown without shaking at 23°C at 100 μmol light intensity, on a 12:12 h light: dark cycle. Strains were grown on BG11‐2N media (recipe available as Data S1), modified to contain 200 μM of NO_3_^−^, NH_4_^+^, or urea as the sole form of N. The pH of the media was adjusted to between 8.0 and 8.2 using 1 M NaOH. Cultures were maintained on NO_3_^−^ and were acclimated to the other two N substrates in 10 mL cultures. 5 mL of NO_3_^−^ culture were added to 5 mL of NH_4_^+^ or urea media and grown for 1 week. 1 mL of the 1:1 mixed culture was then transferred to 9 mL of the new media. Cultures were then scaled up weekly at a 1:10 dilution, first to 50 mL total volume and then to 100 mL. Each strain was transferred in 100 mL volume at least three times before starting the experiment, to minimise any residual NO_3_^−^. Once acclimated to the media, each strain was grown in triplicate in 100 mL of each type of media, for a total of 27 experimental groups and an additional 3 uninoculated media controls.

### 2.3 Collection and Extraction of Extracellular Metabolites

Cultures were harvested in the mid–late exponential growth stage. Given that many *M. aeruginosa* isolates are colonial, tracking growth accurately is sometimes challenging (Yancey, Kiledal, et al. 2023, Große et al. 2024). We estimated growth stage based on growth curves constructed from cell counts during culture acclimation. LE19‐197.1, which grows fastest among the selected strains, was harvested on day 8 post‐inoculation. PCC 7806 was harvested on day 9, and LE19‐10.1 on day 10. Uninoculated controls were harvested on day 9. Fifty mL of spent media from each culture was collected by filtering cells through pre‐combusted 47 mm GFF filters (Millipore‐Sigma). Spent media was frozen, then lyophilized before metabolite extraction. To account for differences in the amount of material filtered, filters were dried at 50°C overnight, and total particulate organic carbon and nitrogen (POC and PON) were analysed using elemental analysis (EA) on a Thermo Scientific Flash EA instrument using a thermal conductivity detector (TCD). These data were used to normalise metabolite abundance between treatments, which differed slightly in POC and C:N ratio (pH of the media was adjusted to between Table S1). Peak areas of metabolites for each treatment group were normalised to the respective POC amounts in order to account for differences in biomass.

Extraction of metabolites from spent media followed procedures established by the Joint Genome Institute (JGI, Louie et al. 2024). This extraction method targets a large range of primary and secondary metabolites, but may not capture metabolites with hydrophobic qualities or those related to the extracellular polymeric substance (EPS) fraction of these cultures. Lyophilized samples were redissolved in 2 mL of 100% methanol and transferred to 4 mL vials, vortexed, then sonicated in an ice bath for 10 min. The vials were centrifuged at 5000 rpm for 5 min, after which the supernatant was transferred to 2 mL vials, dried down under a gentle stream of high purity N_2_ gas at 30°C, and stored frozen. Just prior to mass spectrometry analysis, the dried extract was resuspended in 200 μL of methanol, vortexed, then sonicated in an ice bath for 10 min. Samples were filtered with a mini syringeless filter unit (Whatman, WHAUS503NPEORG), then transferred to LC–MS vials.

### 2.4 Liquid Chromatography‐Mass Spectrometry (LC–MS)

Samples were run on a Vanquish Ultra High Performance Liquid Chromatograph connected to a Thermo Scientific Q‐Exactive Orbitrap mass spectrometer equipped with a heated electrospray ionisation (HESI‐II) source and running in positive ion mode (UPLC–MS). Sample (5 μL) was injected onto an Agilent Poroshell 120 HILIC‐Z column (2.1 × 150 mm, 2.7 μm, 100 Å) held at 40°C and a flow rate of 0.45 mL/min. The HPLC gradient and mobile phases are found in Table S3. MS data were collected over a mass range of 70–1050 m/z, using data‐dependent MS/MS analysis with 0.5‐s dynamic exclusion enabled and stepped fragmentation energy (20, 25, 30 eV). Methanol blanks and uninoculated media controls were run alongside samples, which were run in a randomised order. All data presented in this study can be found on the MASSiVE database at this doi: 10.25345/C5B27Q37C.

### 2.5 Data Processing and Feature‐Based Molecular Networking (FBMN)

**FIGURE 1:**
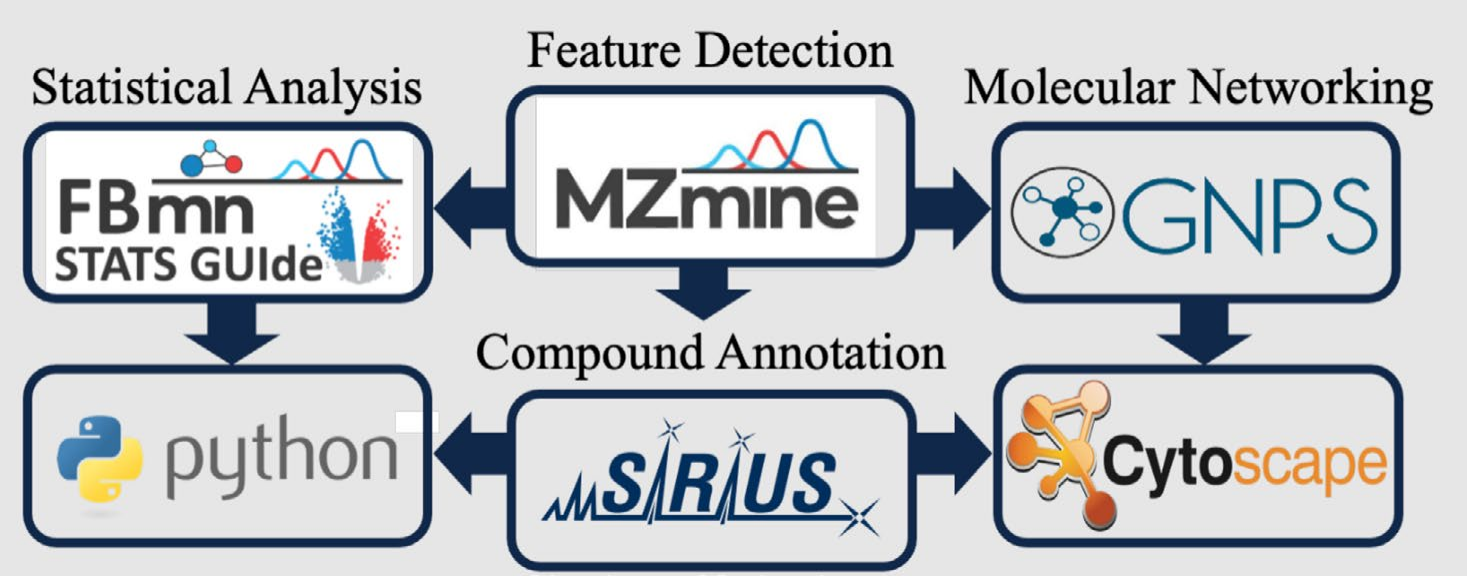
Summary of data analysis workflow. Features were detected via mass spectrometry analysis using MZmine. Compounds were annotated using SIRIUS. Molecular networking was performed in GNPS 1, and the output was visualised in Cytoscape. SIRIUS annotations were added to the Cytoscape network. Statistical analysis was performed in the FBmn STATS GUIde webapp and using Python, where SIRIUS annotations were combined with peak area data.

A schematic of the complete workflow is shown in Figure 1. Raw data files were converted to mzXML file format and imported into MZmine version 3.9.0 (Schmid et al. 2023). Parameters used in the feature‐finding processing wizard were: smoothing checked, stable ionisation across samples checked, RT cropped between 1.30 and 20.50 min, maximum 15 peaks in chromatogram, minimum 4 consecutive scans. Approximate full width at half maximum (FWHM) of 0.1, intra‐sample RT tolerance of 0.01, and sample‐to‐sample tolerance of 0.4 were all evaluated as minimum absolute intensities. Mass detector selected was factor of lowest signal, noise threshold MS1 of 5.00, MS2.MSn of 2.50, minimum feature height of 5.0E6, scan‐to‐scan m/z tolerance of 0.002 m/z or 10 ppm, intra‐sample tolerance of 0.0015 m/z or 3 ppm, and sample‐to‐sample tolerance of 0.0015 m/z or 5 ppm. Original feature list was kept, minimum samples per aligned feature were set at 1 sample or 0%. The options for “apply spectral networking”, “export for molecular networking”, and “export for SIRIUS” were all checked. The processed data were exported as a feature quantification table (gnps_quant.csv), a spectral summary (gnps.mgf), and for SIRIUS analysis (sirius.mgf) for downstream analyses.

The feature quantification table and spectral summary from MZmine were input into the GNPS1 platform (Wang et al. 2016) using the FBMN workflow (Nothias et al. 2020). The network was created with a minimum cosine score of 0.7 and more than 6 matched peaks, with both the precursor and fragment ion mass tolerances set to 0.02 Da. The parameters for the library search were the same, with a score threshold of 0.7 and a minimum 6 matched peaks. A link to the GNPS job can be found here. Of the 14 metabolites identified as library hits in databases available within GNPS (Table 2), two were attributed to contamination from lab plasticware (decaethylene glycol and 13‐docosenamide, Keller et al. 2008) and were not considered in further analyses (see all library matches in Table S2 and mirror matches in Figure S1). One library hit, tyrosine, was only detected in one replicate of LE19‐10.1 grown on NO_3_^−^ and was therefore also not considered in further analyses.

**TABLE 2:** List of library hits detected across treatments, including chemical formula, classy fire most specific class annotation, suspected function in *M. aeruginosa* and shorthand label.

Name	Formula	Most specific class	Schymanski confidence	Function	Label
Microcystin LR	C_49_H_74_N_10_O_12_	Oligopeptides	N/A	Cyanotoxin	A
Aerucyclamide A	C_24_H_34_N_6_O_4_S_2_	Oligopeptides	0.95	Cyanotoxin	B
Anabaenopeptin NZ857	C_45_H_59_N_7_O_10_	Oligopeptides	0.89	Cyanotoxin	C
Shinorine	C_13_H_20_N_2_O_8_	Alpha amino acids and derivatives	N/A	Photoprotectant	D
Porphyra‐334	C_14_H_22_N_2_O_8_	Alpha amino acids and derivatives	0.93	Photoprotectant	E
β‐Carotene	C_40_H_56_	Lipids and lipid‐like molecules	0.84	Photoprotectant	F
Ergothioneine	C_9_H_15_N_3_O_2_S	Histidine and derivatives	0.99	Antioxidant	G
3‐Deazauridine	C_10_H_13_NO_6_	Pyrimidine nucleosides	0.99	Antiviral	H
5′Methylthio‐adenosine	C_11_H_15_N_5_O_4_S	5′‐deoxy‐5′thionucleosides	0.99	Methionine salvage	I
L‐Tryptophan		Indoles and derivatives	0.94	Amino acid	J
Arginine		Alpha amino acids and derivatives	0.99	Amino acid	K

*Note:* “Most specific class” refers to the lowest level of classification that could be assigned based on an 80% confidence threshold (see Section 2). See Figure 5 for the relative abundance of each metabolite in individual treatments.

Molecular network visualisation was performed in Cytoscape version 3.10.1 (Shannon et al. 2003). Network nodes correspond to metabolites, while the edges between them represent cosine scores calculated by comparison of mass spectra; nodes connected via edges are therefore significantly structurally related to each other. Metabolites excluded from previous statistical analysis due to their presence in methanol blanks and/or uninoculated controls were removed from the Cytoscape network. Nodes were coloured according to ClassyFire SuperClass annotation (see below) and were scaled relative to the sum of all peak areas of the treatments in which they were detected (Mannochio‐Russo et al. 2022).

### 2.6 Prediction of Chemical Formulas and Compound Classification via SIRIUS

In addition to using FBMN and GNPS to annotate molecular features with spectral library matching, we used SIRIUS version 5.8.3 (Dührkop et al. 2019) to predict the molecular formulae, structures, and compound classes of metabolites from their mass spectra. SIRIUS is a software framework that integrates multiple machine learning tools for metabolomics. SIRIUS assigns molecular formulas to spectra using fragmentation trees and isotope pattern analysis. ZODIAC then analyses fragmentation trees to re‐rank formula candidates, taking the characteristics of other compounds in the dataset into account (Ludwig et al. 2020). Fragmentation trees were also input into CSI: FingerID to predict molecular fingerprints for each compound (Dührkop et al. 2015).

CANOPUS was applied to the predicted structures to assign them a ClassyFire chemical taxonomy (Djoumbou Feunang et al. 2016). This taxonomy, from most to least broad, includes superclass, class, and subclasses of organic compounds. “Superclass” includes 26 categories of compounds with general structural identifiers (e.g., organic acids and derivatives, phenylpropanoids and polyketides), “class” includes 764 categories of compounds with more specific and recognisable structural features (e.g., pyrimidine nucleosides, carboxylic acids and derivatives, organonitrogen compounds), and “subclass” includes 1729 categories of compounds (e.g., amino acids, peptides, and analogs, fatty acids and conjugates). Using this classification system, microcystin‐LR would have the following categorisation: superclass = Organic acids and derivatives; class = Carboxylic acids and derivatives, subclass = Amino acids, peptides, and analogues. CANOPUS predicts confidence scores for each taxonomic classification; in this study, designations below 80% confidence were not accepted unless a metabolite was linked to others assigned at higher confidence within the exometabolome molecular network (Figure S2).

To establish an appropriate threshold for annotation confidence, we examined SIRIUS annotations of library hits from the Global Natural Product Social Molecular Network database (GNPS; Wang et al. 2016). All library hits were assigned at the superclass level by SIRIUS with at least 80% confidence except for shinorine, which was classified as a benzenoid at 60% confidence. However, the structure of shinorine does not contain a benzene ring; shinorine was therefore manually reclassified as a member of the organic acids and derivatives category, matching its analog, porphyra‐334. All other metabolites (without library hits) annotated with SIRIUS at less than 80% confidence at the superclass level were then manually reassigned to the “Unassigned” category, unless they were linked to other metabolites in the molecular network that were annotated at a higher confidence. In the latter case, metabolites were manually assigned to match the superclass of their associated molecular families. 248 metabolites, including the library hits, were therefore annotated at the superclass level or below. Out of the original 274 metabolites identified, 165 metabolites had sufficient structural information to be annotated at the subclass level at 80% confidence or above. In general, metabolite identifications in this study, including GNPS library hits, should be considered putative, as advanced structural determination (e.g., compound isolation, comparison with authentic standards, structural determination via NMR) was not performed. GNPS library hits are considered level 3 confidence matches, while SIRIUS annotated features are considered level 4 confidence matches based on the Schymanski et al. (2014) classification scheme.

### 2.7 Statistical Analysis

Statistical analysis described below followed the workflow and code in Pakkir Shah et al. (2023) using the web interface for the Functional Metabolomics Lab's FBMN‐STATS Guide (https://fbmn‐statsguide.gnps2.org/). The feature quantification table generated in MZmine and metadata were imported into FBMN‐STATS. Background features were eliminated by first dividing the sampling groups into controls and experimental treatments. The average peak areas of each feature within the controls and experimental treatments were calculated and a control‐to‐experimental treatment ratio for each feature was obtained by dividing the average peak area in the controls by the average peak area in the experimental samples. This ratio was compared to a user‐defined cutoff of 0.1 to determine which features to retain (as recommended in Pakkir Shah et al. 2023). The selected cutoff of 0.1 meant a particular molecular feature could have up to 10% of its peak area from controls and 90% from experimental treatments. If the feature's control‐to‐experimental treatment ratio exceeded 0.1, it was removed as a likely background feature. To account for differences in the amount of biomass between treatments, peak areas were first normalised to each treatment's corresponding POC value. Missing values were imputed, and data were normalised via centre‐scaling (achieved by subtracting the mean peak area of each metabolite from the peak area values for said feature that were obtained from each sampling group, then dividing the centered metabolites by their standard deviations, see Pakkir Shah et al. 2023). This yielded data that were centred around 0 and compensated for inconsistencies caused by peak area differences between metabolites with high abundance versus low abundance (Pakkir Shah et al. 2023).

Principle Coordinate Analysis (PCoA) was performed using the Bray‐Curtis distance metric to compare the relative impacts of strain and N substrate type on the metabolome. The top two principal coordinates were visualised using the FBMN‐STATS Guide web interface. Permutational multivariate ANOVA (PERMANOVA) was also performed on culture and media distance matrices to test for clustering significance.

Center‐scaled peak area data from experimental treatments were arranged into a dendrogram via hierarchical clustering with a Euclidean distance matrix and a complete linkage matrix. A heatmap of relative metabolite abundances across experimental treatments was generated, with columns representative of each treatment, and arranged according to hierarchical clustering pattern. Rows represent metabolites and were grouped by superclass, then class, then arranged according to Euclidean distance of detection pattern. This visualisation enabled comparison of the relative abundance and chemical categories of exometabolites driving the treatment differences seen in the PCoA, and also revealed internal variability within replicates. Annotations from SIRIUS and the GNPS spectral library were combined with feature table data in Python to analyse the chemical composition of both scaled and unscaled peak area data.

To compare differences among the most abundant compounds produced under each growth condition, we filtered the center‐scaled data to only include metabolites with highly above‐average peak areas. We set this threshold at the highest 75th percentile value (Q3) observed among all treatment groups (see Figure S4). Metabolites with center‐scaled peak areas greater than 1 were retained for this portion of our analysis. All code to produce the analyses and figures in this paper can be found here: https://github.com/cmpeck01/Peck‐et‐al.‐2025/tree/main.

## 3 Results

### 3.1 Chemical Diversity of the *M. aeruginosa* Exometabolome

**FIGURE 2:**
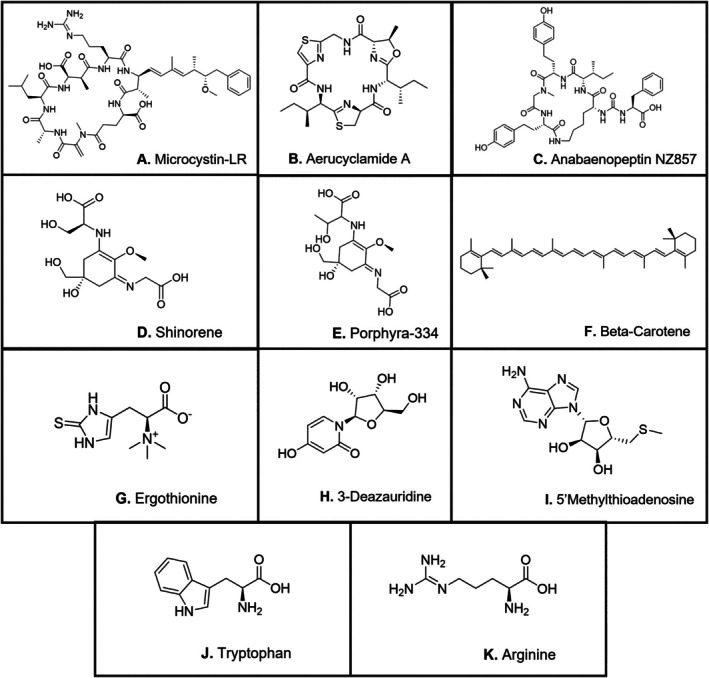
Structures of GNPS library hits listed in Table 2. Mirror matches showing spectral similarities are in Figure S1.

274 metabolites were detected across all cultures and N treatments after filtering out background features (Figure S2). Only 11 metabolites matched spectra from previously characterised compounds available in GNPS structural databases (Wang et al. 2016). 248 metabolites were annotated with ClassyFire at the superclass level or below, and out of these, 165 metabolites were annotated at the subclass level (Dührkop et al. 2019). Table 2 presents a summary of information on compounds that putatively matched GNPS library spectra, with structures shown in Figure 2. Mirror plots showing matching peaks between library and experimental spectra for the library hits in Table 2 are shown in Figure S1.

The largest compound superclasses in the exometabolomes were organic acids and their derivatives, and organoheterocyclic compounds. The organic acids and derivatives superclass was primarily composed of N‐containing metabolite subclasses such as amino acids, peptides, and analogues, with a few metabolites annotated as organosulfonic acids and derivatives, phosphate esters, beta hydroxy acids and derivatives, dicarboxylic acids and derivatives, and carboxylic acid derivatives. Metabolites in the organoheterocyclic compounds superclass were primarily identified as purines/purine derivatives and pyrimidines/pyrimidine derivative subclasses, with a few instances of pterin‐like metabolites, quinolones and derivatives, pyridoxines, pyrazines, pyridinecarboxylic acids and derivatives, and imidazoles.

### 3.2 Exometabolome Composition Differs Significantly in Cultures Grown on NO_3_ ^−^ Versus NH_4_ ^+^ or Urea

**FIGURE 3:**
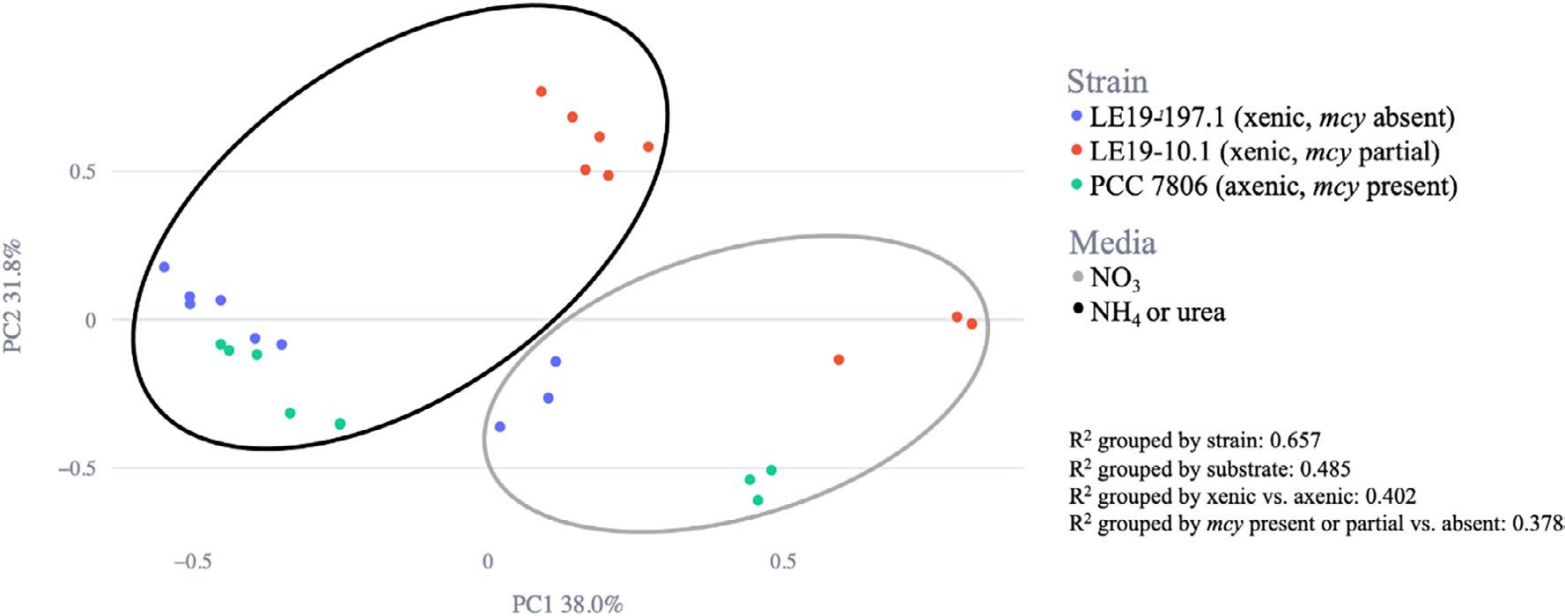
PCoA plot of centre‐scaled peak area data, with points representing experimental groups. Points are coloured according to strain and grouped to illustrate clustering patterns between N substrate treatments.

Principle coordinate analysis (PCoA) of the center‐scaled peak area data showed that variations in exometabolome composition are driven by N substrate and *M. aeruginosa* strain (Figure 3). NO_3_^−^ grown cultures are distinct from NH_4_^+^ and urea‐grown cultures, separated by principal coordinate 1 on the *x*‐axis. In terms of strain, when grown on NO_3_^−^ each strain clusters separately from the others, but when grown on NH_4_^+^ and urea, PCC 7806 and LE19‐197.1 overlap, and only LE19‐19.1 clusters separately. PERMANOVA analysis confirmed statistical significance of this clustering based on both strain and N substrate (*p* values ≤ 0.001). In addition, *R* ^2^ values of strain‐grouped PERMANOVA and substrate‐grouped PERMANOVA were 0.619 and 0.509, respectively, demonstrating that N substrate differences explained almost as much of the observed variance in metabolite abundance as strain differences. The *R* ^2^ value of xenic vs. axenic PERMANOVA was 0.370, and *Mcy* present or partial vs. absent was 0.384, showing that in comparison to these two factors, N substrate was a greater predictor of variance in metabolite abundance.

**FIGURE 4:**
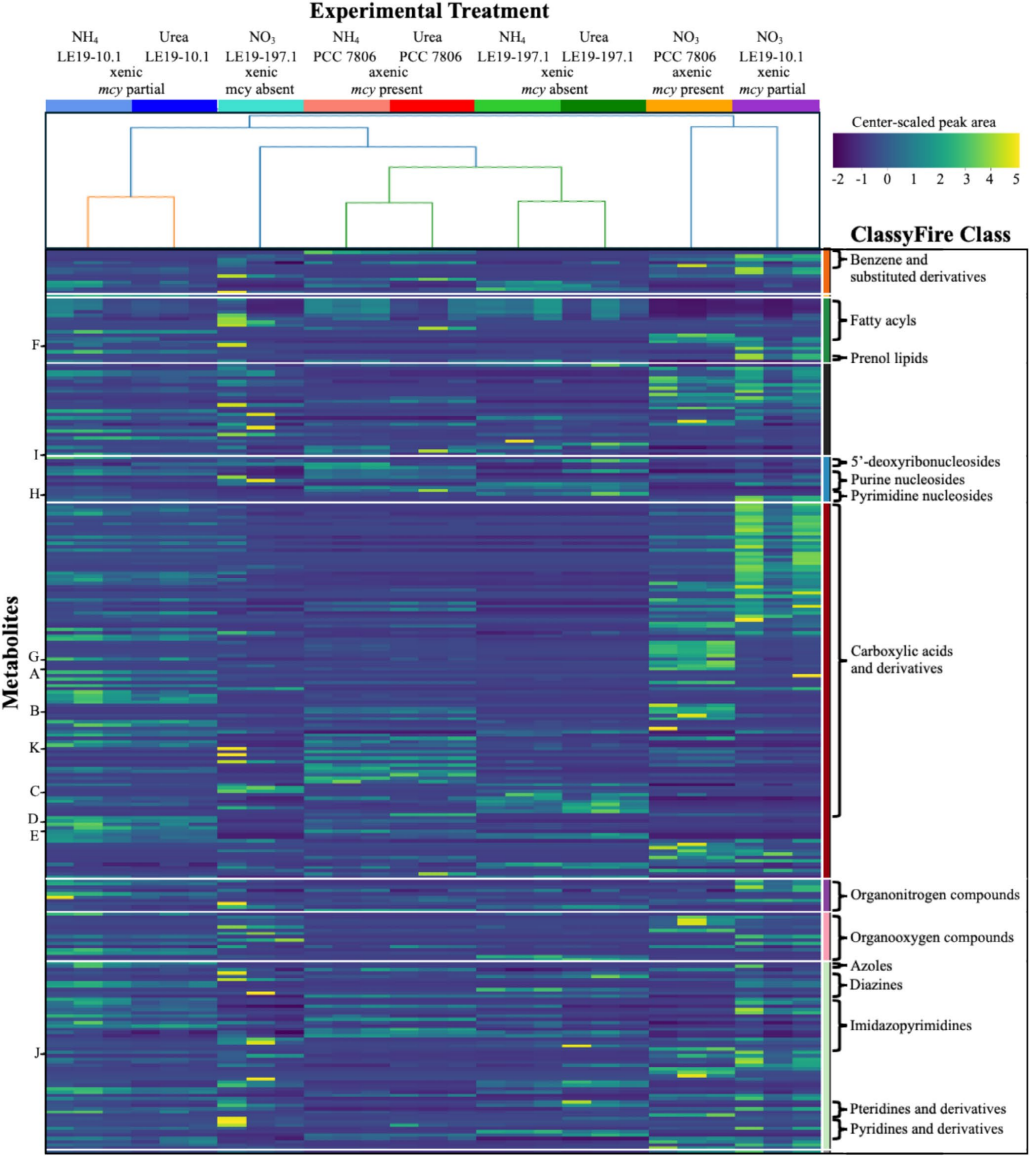
Dendrogram, coloured based on degree of similarity between treatment groups (euclidean), and heatmap of center‐scaled peak area data. Negative values on the heatmap represent below average levels of abundance of an exometabolite, while positive values represent above average levels of abundance. Each leaf of the dendrogram corresponds to three columns on the heatmap, which are grouped together based on experimental treatment (three biological replicates per treatment). The columns were ordered according to the clustering pattern of the dendrogram. Each row corresponds to an exometabolite, which were grouped vertically by superclass, then class, then arranged according to Euclidian similarity of feature detection patterns across treatments (Table S5 contains classification information on each metabolite, arranged in the same order). The vertical colour bar corresponds to the ClassyFire superclasses of each metabolite, with colours assigned in accordance with Figure S2 (from top to bottom, orange = benzenoids, green = lipids and lipid‐like molecules, black = unassigned, blue = nucleosides, nucleotides, and analogues, red = organic acids and derivatives, purple = organic nitrogen compounds, pink = organic oxygen compounds, pale green = organoheterocyclic compounds). Brackets show location of ClassyFire classes within each superclass. Library hits are notated with letters. The colour bar at the top of the figure corresponds to the colours of each treatment as presented in Figure 5.

Exometabolome compositions from the samples were hierarchically clustered and arranged based on Euclidean distance metrics and a complete linkage matrix, to reveal specific patterns of exometabolite variation underlying the differences between NO_3_^−^ ‐grown cultures and NH_4_^+^ or urea‐grown cultures (Figure 4). The exometabolomes of PCC 7806 and LE19‐197.1 were more similar in NH_4_^+^ and urea treatments compared to LE19‐10.1, but when grown on nitrate, PCC 7806 was more like LE19‐10.1 than LE19‐197.1. However, the smallest unit of clustering contains only one strain and N substrate treatment, demonstrating that the exometabolomes under NH_4_^+^ and urea enrichment, despite their resemblance to one another, remained distinct enough to be distinguished via hierarchical clustering.

### 3.3 N Substrate Influence on Exometabolite Diversity

To examine differences in the composition of the most abundant molecules between N substrate treatments, center‐scaled data were filtered for each N substrate treatment to only include metabolites with highly above‐average peak areas (see Figure S4), which we henceforth refer to as “highly abundant metabolites”. Only metabolites with center‐scaled peak areas greater than 1 were considered for this portion of our analysis.

Of the metabolites detected at highly above‐average levels, amino acids, peptides, and analogues were the most common compound subclass detected across all treatments, comprising from 20.0% (LE19‐197.1 NH_4_^+^) up to 60.0% (PCC 7806 NO_3_^−^) of the highly abundant metabolites from each treatment, with five of the treatments containing at least 40% amino acids. Fatty acids and conjugates comprised from 1.8% (PCC 7806 NO_3_^−^) up to 33.3% (LE19‐197.1 NH_4_), with four of the treatments containing less than 5% fatty acids. Purines and purine derivatives comprised between 0% (LE19‐10.1 urea, LE19‐197.1 NH_4_) and 16.0% (PCC 7806 urea), with four of the treatments comprised of over 10% purines. Unlike amino acids, neither fatty acids nor purines showed uniform patterns of high relative abundance across all treatments (Table S4, Figure S3).

Strains grown on NO_3_^−^ exhibited the most diverse exometabolome, containing more compound subclasses detected at highly above‐average levels compared to their NH_4_^+^ and urea counterparts. PCC 7806 grown on NO_3_^−^ led to detection of the most subclasses of these metabolites: 55 compounds across 20 different compound subclasses. In contrast, the exometabolomes of PCC 7806 grown on NH_4_^+^ and on urea contained only seven and eleven compound subclasses, respectively (Table S4, Figure S3). Twenty‐four of the highly abundant compounds were produced exclusively in cultures grown on NO_3_^−^. These were mostly compounds classified as amino acids, with one compound each of pyrimidines, pterins, guanidines, and benzenetriols, and seven compounds that were unassigned at the subclass level.

Only three compounds were exclusively detected at highly above‐average levels in cultures grown on NH_4_^+^ or urea, all of which were amino acids. No highly abundant compounds were produced in growth on NH_4_^+^ that were not also produced on urea and vice versa. In LE19‐197.1, culturing with urea yielded the highest number of metabolites that were detected in greater abundances than in other treatments. In LE19‐10.1 and PCC 7806, culturing with NO_3_^−^ resulted in the greatest number of metabolites detected at highly above‐average levels. In contrast, the lowest number of highly above‐average metabolites was detected in urea treatments for LE19‐10.1 and in NH_4_^+^ treatments for PCC 7806.

### 3.4 Relative Abundance of Library‐Identified Compounds Varied Between N Substrate Treatments

**FIGURE 5:**
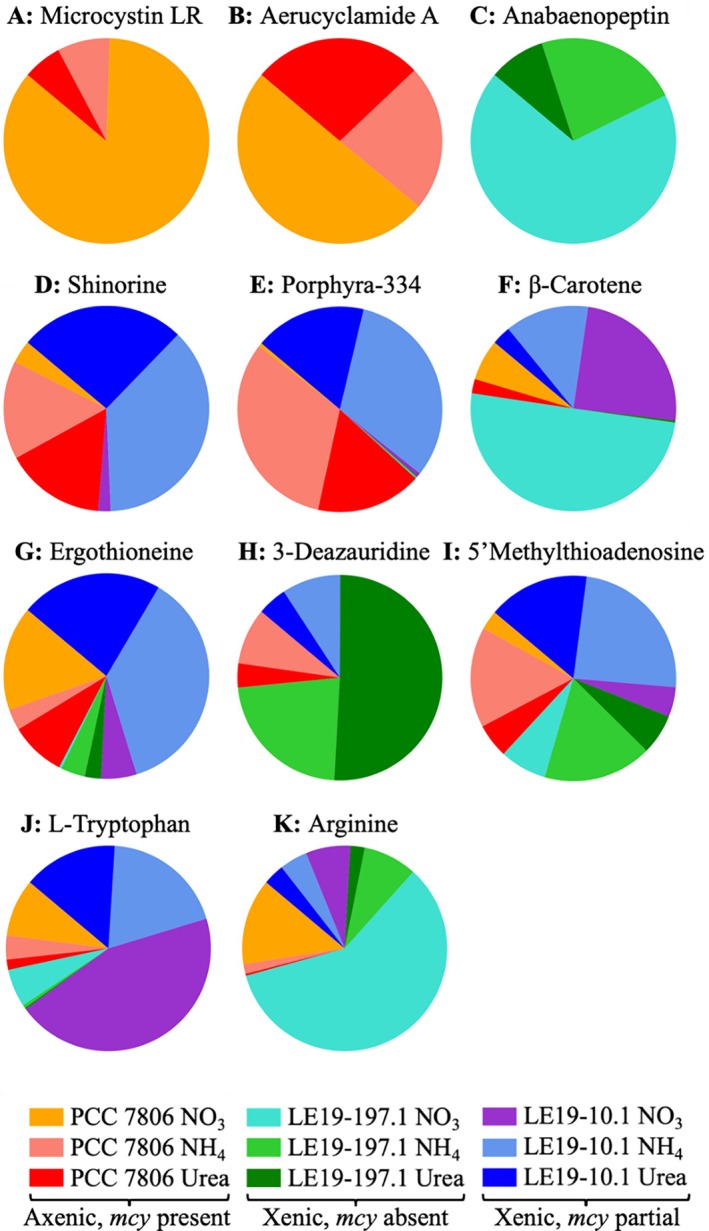
Relative abundance levels of library‐identified metabolites across treatments. Colours of treatments are also used in Figure 4 to indicate their order in the dendrogram.

The relative amounts of metabolites with library spectral matches under each N substrate treatment were compared using unscaled feature peak area data (Figure 5). Compounds putatively identified as cyanotoxins microcystin‐LR (Figure 2A) and aerucyclamide A (Figure 2B) were only produced by PCC 7806 and were detected at the highest abundance in NO_3_^−^ treatments. Another hepatotoxin from cyanobacteria, anabaenopeptin NZ857 (Figure 2C), was only detected in LE19‐197.1 cultures. While this compound was detected in all N substrate treatments for LE19‐197.1, it was detected at the highest abundance in NO_3_^−^ ‐grown cultures.

Some compounds involved in photoprotection or oxidative stress exhibited N substrate‐specific patterns of abundance. Two structurally related microbial sunscreen compounds (Soule and Garcia‐Pichel 2013), shinorine (Figure 2D) and porphyra‐334 (Figure 2E), exhibited an opposite pattern compared to microcystin‐LR and aerucyclamide A, where they were produced by both PCC 7806 and LE19‐10.1 at higher abundances in NH_4_^+^ or urea‐grown cultures compared to NO_3_^−^. Porphyra‐334 was detected only in LE19‐197.1. β‐carotene (Figure 2F), a photoprotective pigment (e.g., Zhu et al. 2010), was detected at highest relative abundance in strains grown on NO_3_^−^, with PCC 7806 grown on urea the only treatment where it was not detected. Ergothionene (Figure 2G), an antioxidant (Hartman 1990), was detected primarily in PCC 7806 grown on NO_3_^−^ and LE19‐10.1 grown on NH_4_^+^ or urea. It was detected in smaller amounts in all other sample groups.

3‐deazauridine (Figure 2H), a pyrimidine analog with antiviral activity (Khare et al. 1972), was only detected under NH_4_^+^ and urea enrichment in all strains. 5′methylthioadenosine (Figure 2I), an intermediate in the methionine salvage pathway (Albers 2009), exhibited similar relative abundances across all sample groups, with slightly higher abundances under NH_4_^+^ or urea treatment across all strains. A nucleotide‐based compound, the antiviral 3‐deazauridine (Shannon et al. 1972), was not previously identified in *M. aeruginosa* and was only detected in NH_4_^+^ and urea treatments. This was the only library hit that was detected in all strains, but only during growth on reduced N substrates.

Variability in the prevalence of these metabolites across treatment groups is apparent in Figure 4, with microcystin forming part of the cluster of positive center‐scaled peak areas found under the PCC 7806 NO_3_^−^ column within the “Carboxylic acids and derivatives” class. Shinorine and porphyra‐334 form a distinct cluster most visible within the LE19‐10.1 NH_4_^+^ and urea columns, while ergothionene overlaps with both this cluster and the microcystin cluster, showing elevated levels of production under the PCC 7806 NO_3_^−^, LE19‐10.1 NH_4_^+^, and LE19‐10.1 urea treatments (Figure 4).

## 4 Discussion

This study characterised the diversity of the *M. aeruginosa* exometabolome and its response to changes in N substrate. Across all exometabolomes, the most common compound subclasses detected were N‐containing molecules, including amino acids, purines, and pyrimidines. Amino acids were detected at high relative abundance across all treatments (comprising 19%–50% of all compounds detected at above background levels in each treatment), which is consistent with the central role of amino acids in cellular N assimilation and metabolism (Muro‐Pastor et al. 2005; Esteves‐Ferreira et al. 2018). This also agrees with previous work that identified amino acids as a major component of *M. aeruginosa* exudate (Yan et al. 2023).

Because we analysed spent media, the detection of any given metabolite may be due to either export from living *M. aeruginosa*, release after cell death, or contributions from associated heterotrophic bacterial communities. Many of the library hits, including cyanotoxins, have poorly characterised export dynamics, and data are inconclusive regarding the existence and efficiency of transporters capable of moving these compounds into the extracellular space (Neilan et al. 2013; Pearson et al. 2004). However, any of these mechanisms resulting in the release of metabolites from cells would occur in both natural communities and cultures and are therefore relevant to understanding phycosphere dynamics and microbial interactions.

### 4.1 The Influence of N Substrate on Exometabolome Composition and Structural Diversity

This study identified the highest similarity between the exometabolomes of *M. aeruginosa* strains grown on reduced N substrates, and dissimilarity to cultures grown on NO_3_^−^. Strains grown on NO_3_^−^ also exhibited the most diverse exometabolomes (in terms of numbers of compounds detected across distinct molecular subclasses), when compared to strains grown on either NH_4_^+^ or urea. This suggests that the exometabolome fingerprint is influenced by the thermodynamics and bioenergetics of N assimilation, as suggested previously for intracellular metabolites (Krausfeldt et al. 2020).

In cyanobacteria, C and energy metabolism are connected to N metabolism via a highly conserved N regulatory system, which senses cellular N status through the balance between intracellular C and N levels (Esteves‐Ferreira et al. 2018). The regulation and energy expense involved in assimilating different N substrates could therefore result in altering N flux through downstream metabolic pathways in cells, affecting the exometabolome as well. As the most reduced form of N, NH_4_^+^ is thought to be “preferred” by most phytoplankton over other N substrates, because NH_4_^+^ requires less energy to assimilate into cellular biomass (Flores and Herrero 2005; Veaudor et al. 2019). In contrast, NO_3_^−^ requires more energy and cellular machinery for cross‐membrane transport, and must be reduced to NO_2_^−^ before it is converted to NH_4_^+^, a process that requires four times the amount of energy compared to NH_4_^+^ assimilation (Glibert et al. 2016; Muro‐Pastor et al. 2005). This increased demand for carbon and energy is supported by the higher C:N ratios observed in NO_3_^−^‐grown cultures compared to cultures grown on reduced N substrates (Table S1).

At the molecular level, NH_4_^+^ and urea are both reduced forms of N which should be energetically less expensive for cells to assimilate and could result in similar exometabolite production. However, urea metabolism differs from NH_4_^+^ as it is hydrolysed to form two molecules of NH_4_^+^ after import into the cytosol (Flores and Herrero 2005), and *M. aeruginosa* can also use urea as a source of both N and C (Krausfeldt et al. 2019). Because urea provides twice the amount of N and an additional C source, recent studies have suggested that urea assimilation may lead to greater ATP production and reducing power, and production of different metabolites, compared with growth on NH_4_^+^ (Krausfeldt et al. 2020).

In addition to redox energetics, another possible reason for the similarity between the exometabolites of NH_4_^+^ and urea‐grown cultures is that, under high N concentrations, excess NH_4_^+^ produced through urea hydrolysis may be excreted back into the environment (Chen et al. 2019; Sakamoto et al. 1998), which over time could effectively transform the urea substrate treatment into a mix of urea and NH_4_^+^.

### 4.2 Strain‐Specific Effects on Exometabolome Composition

Most of the strain‐specific characteristics (e.g., toxin production, axenic/xenic, isolation location/date, see Table 1) were not reflected in clear patterns of differential metabolite relative abundance. For example, in NH_4_^+^ and urea treatments, the exometabolome composition of PCC 7806 and LE19‐197.1 clustered together, even though both LE19‐197.1 and LE19‐10.1 were collected from similar geographical areas and time points, and both contain heterotrophic bacteria (Yancey, Kiledal, et al. 2023), while PCC 7806 does not. Similar patterns in the relative abundance of microcystin LR and aerucyclamide A in PCC 7806, and of anabaenopeptin NZ857 in LE19‐197.1 under each N treatment also suggest that bacteria with cyanotoxin‐degrading capabilities imparted minimal effects on the detection of cyanotoxins in the exometabolomes of xenic strains. On the other hand, phylogeny and genetic variation between strains likely do affect strain‐dependent responses to different N substrates. Phylogenetically, LE19‐197.1 is more closely related to PCC 7806 than to LE19‐10.1 (Yancey, Kiledal, et al. 2023). However, PCC 7806 and LE19‐10.1 are most similar in terms of microcystin production capabilities, with PCC 7806 possessing a complete *mcy* operon (Frangeul et al. 2008) and LE19‐10.1 possessing a partial *mcy* operon (Yancey, Kiledal, et al. 2023). LE19‐197.1 possesses no *mcy* operon and cannot produce microcystin (Yancey, Kiledal, et al. 2023). The composition of above‐average metabolites in PCC 7806 and LE19‐10.1 cultured on NO_3_^−^ was very similar (Table S4, Figure S3), but less so when grown on other N substrates. This could indicate that there are more strain‐level metabolic similarities for utilisation of NO_3_^+^ than for NH_4_^+^ and urea. In general, *M. aeruginosa* strains (including the strains used in this study) have very similar genetic capabilities for N assimilation and transport (Yancey et al. 2022; Dick et al. 2021), and therefore any differences in utilisation of N substrates between strains are likely caused by strain‐specific differences in gene expression or in the kinetics of N assimilation machinery.

### 4.3 Ecological Implications of *M. aeruginosa* Exometabolome Composition

Results of this study suggest that changes in the types of N available during the course of a bloom can affect the compounds released into the environment by *M. aeruginosa*, which could be related to observed shifts in *M. aeruginosa* strain and phycosphere community composition in the field (Smith et al. 2021). In Lake Erie, the most abundant N substrate through much of the peak bloom season is NO_3_^−^, which many bacteria are unable to use, but which *M. aeruginosa* can assimilate. Under these conditions, bacteria may gain an advantage by associating with the *M. aeruginosa* phycosphere to access N‐containing exudates. As N availability decreases through the bloom, with the N pool shifting from primarily NO_3_^−^ to mostly organic forms of N, the role of bacteria may also shift, with some competing with *M. aeruginosa* for NH_4_^+^ and urea (Li et al. 2024), while others may facilitate N uptake through recycling of dipeptide and oligopeptides produced by *M. aeruginosa* or other N‐containing compounds (Smith et al. 2022).

This study and previous work (Yan et al. 2023) identified N‐containing molecules, especially amino acids and peptides, at high relative abundance across all strains and N treatments. This results in a wide variety of N‐containing compounds that phycosphere‐associated bacteria may be able to use, depending on their specific metabolic capabilities. Amino acids are important sources of N for many bacteria that are often derived from phytoplankton (e.g., Ferrer‐González et al. 2021; Tupas et al. 1994). The release of amino acids from *M. aeruginosa* has a wide range of proposed ecological effects that include facilitating bacterial colonisation, providing substrates for ammonification, and retaining N by promoting N recycling processes rather than denitrification (Yan et al. 2023). Previous metatranscriptomics work also showed that genes putatively encoding amino acid, peptide, and nucleoside uptake and transport were some of the most highly expressed genes in bloom‐associated heterotrophic bacteria during *M. aeruginosa* blooms (Smith et al. 2022). Detectable expression of amino acid oxidases and peptidases also suggests that bacteria can deaminate released amino acids or break down oligopeptides to access N during *M. aeruginosa* blooms (Smith et al. 2022), which may help recycle organic N into forms that *M. aeruginosa* can use.

Unlike amino acids, compounds annotated as purines and pyrimidines did not follow a uniform pattern of high relative abundance across treatments. This compound subclass was previously identified in *M. aeruginosa* exudate, and its relative abundance varied between low and high‐microcystin producing strains (Zhou et al. 2023). In other cyanobacteria, they function in signalling, regulation, and/or light‐harvesting during light sensing and adaptation processes (Moon et al. 2012). Purines and their derivatives participate directly in N incorporation via their *de novo* synthesis pathways' requirement of glutamine (Moffatt and Ashihara 2002); however, their relative abundances did not show obvious patterns across N substrate conditions.

N substrate availability may also affect the abundances of non‐N‐containing compounds, such as lipids. The majority of differentially detected lipids in our study were putatively identified as long chain fatty acids. Past research identified differences in lipid metabolism between low and high microcystin‐producing *M. aeruginosa* strains (Zhou et al. 2023), with low microcystin‐producing strains exuding a greater concentration of lipids than high microcystin‐producing strains. Fatty acids do not participate directly in N metabolism, but their synthesis is influenced by the C:N balance and regulatory network within cells, where excess photosynthetic products not used for growth or N assimilation can be stored as fatty acids (Esteves‐Ferreira et al. 2018). Differences across treatments could be due to changes in these coupled metabolic pathways with changes in N substrate, as seen in PCC 7806 where fatty acids are a much lower percentage of differentially abundant metabolites during growth on NO_3_^−^ vs. growth on urea and NH_4_^+^ (2.3% vs. 20% and 25%, respectively, Table S4, Figure S3). However, other strains exhibited much less variation between treatments, suggesting that strain‐specific characteristics may also influence synthesis and extracellular release of fatty acids; for example, some unsaturated fatty acids can inhibit the growth of other species of algae (Ikawa et al. 1996), and thus may be part of a competitive strategy. Lipid metabolism may also be important in oxidative stress management, as work on volatile exometabolites produced in an *M. aeruginosa‐*dominated CyanoHAB showed a positive association between the abundance of microcystin and saturated fatty acid aldehydes, which can be converted to saturated fatty acids in order to repair membrane lipids damaged by oxidative stress (Collart et al. 2023).

### 4.4 N Substrate May Influence Fitness‐Related Strategies in *M. aeruginosa*

Several compounds with functions proposed to influence *M. aeruginosa* fitness were promoted on different N substrates, providing insight into adaptations used by *M. aeruginosa* to balance growth, energy acquisition, and defence under various environmental conditions. Compounds putatively identified as cyanotoxins (Figure 2A–C) and the mycosporine‐like amino acids (MAAs), shinorine and porphyra‐334 (Figure 2D,E), displayed an inverse pattern in relative abundance when grown on different N substrates. Microcystins may aid *M. aeruginosa* in adapting to high light levels and protect against oxidative stress (Guljamow et al. 2021; Meissner et al. 2015; Zilliges et al. 2011, Sakai et al. 2009; Kardinaal et al. 2007). This would give microcystin a similar function to MAAs, which are UV protectants with antioxidant activity (Hu 2022; Whittock et al. 2021; Gao and Garcia‐Pichel 2011). In contrast, aerucyclamide A and anabaenopeptin NZ857 have not been demonstrated to exhibit any photoprotectant qualities (Monteiro et al. 2021; Portmann et al. 2008). Microcystins are also used for grazer defence (Ladds et al. 2021; Bojadzija Savic et al. 2020), with larger amounts of extracellular microcystins associated with larger colony sizes (Gan et al. 2012). Shinorine may also contribute to the formation and integrity of the extracellular matrix and cell–cell interactions within colonies, as it was found exclusively located in the extracellular “slime layer” of *M. aeruginosa* (Hu et al. 2015).

Our results show that the production of microcystin and MAAs could be a compensatory process, with the former acting as a greater photoprotectant in NO_3_^−^ ‐grown cultures, and the latter dominating during growth on NH_4_^+^ and urea. Knockout experiments demonstrated that the lack of shinorine in PCC 7806 did not inhibit growth (when grown on NO_3_^−^, Hu et al. 2015), but this finding might be explained if microcystin, rather than shinorine, were acting as the primary photoprotectant (Hu et al. 2015). In our study, this dynamic between microcystin and MAA production was observed in PCC 7806, which contains a complete *mcy* operon. Due to its partial *mcy* operon, LE19‐10.1 did not produce complete molecules of microcystin (and we were unable to detect the predicted tetrapeptide product, Yancey et al. 2024), but it did produce shinorine and porphyra‐334 under the same N conditions as PCC 7806. On the other hand, LE19‐197.1, which lacks the *mcy* operon, showed minimal production of MAAs, but it produced the UV‐absorbing carotenoid pigment β‐carotene in higher relative abundance when grown on NO_3_^−^ compared to other N substrates.

These results support the hypothesis that N substrate availability may influence oxidative stress management strategies employed by *M. aeruginosa* during blooms (Hellweger et al. 2022), and suggest that more extracellular microcystins may be produced when NO_3_^−^ is the most abundant N substrate during the early and peak bloom stage, whereas more MAAs may be produced later in the bloom when the most available N is recycled NH_4_^+^ or DON. Extracellular MAA concentrations have not been examined in the field, but the highest concentrations of dissolved microcystin typically correspond to the period when NO_3_^−^ is abundant (Gobler et al. 2016).

### 4.5 Implications for Toxin Production

Studies on the effect of N substrate on toxin production have so far produced conflicting results. In this study, microcystins were most commonly detected in the exometabolomes of cultures grown on NO_3_^−^. This agrees with some previous work that shows the highest intracellular microcystin concentrations associated with growth on nitrate (Wagner et al. 2021), but is contradictory to other work, which observed both higher amino acid synthesis and extracellular toxin production in cultures grown on NH_4_^+^ relative to both urea and NO_3_^−^ (Chen et al. 2019). In addition, extracellular toxin concentrations may not necessarily parallel intracellular concentrations: in another study, growth on NO_3_^−^ led to increased intracellular microcystin compared to NH_4_^+^, with the reverse pattern observed in extracellular microcystin concentrations (Yang et al. 2022).

Little research has been done on the relationship between N supply and cyanotoxins other than microcystin, but this study provides evidence that their production may increase under the same conditions that cause an increase in microcystin LR. Aerucyclamide A and anabaenopeptin NZ857 were also detected at greater relative abundance in NO_3_^−^ treatments of PCC 7806 and LE19‐197.1, respectively. This could mean that the bloom stages when microcystin is most abundant (typically mid‐July through early August, when NO_3_^−^ is still present) may also contain higher extracellular concentrations of other cyanotoxins, including those that are currently unmonitored. However, these results conflict with work showing a negative correlation between NO_3_^−^ and expression of certain biosynthetic gene clusters encoding putative cyanotoxins (Yancey, Yu, et al. 2023). There may thus be other environmental controls or fitness trade‐offs between energy and nutrient acquisition that are present in natural communities but not necessarily captured by our experiments with cultured strains. This could include environmental factors like light levels, or biological factors like competition for resources with other phytoplankton or bacteria.

## 5 Conclusions

This study supports the hypothesis that assimilation of different N substrates affects the composition of exometabolites produced in different strains of *M. aeruginosa*. The exometabolomes of *M. aeruginosa* grown on reduced N substrates (NH_4_^+^ and urea) were more similar to each other than to strains grown on NO_3_^−^. This pattern was primarily driven by differences in extracellular amino acids and analogues, fatty acids, and purine derivatives, and likely reflects shifts in metabolic pathways due to differences in N assimilation energetics. Many compounds detected were unique to NO_3_^−^ treatments, while there were no unique compounds produced on NH_4_^+^ or urea. The exometabolomes also provide molecular evidence of fitness‐related adaptations in *M. aeruginosa* such as oxidative stress management, that varied between both strains and N substrate treatments, supporting *M. aeruginosa* as a highly adaptable organism to environmental change. While the relative abundances across treatments of some library‐identified molecules appeared influenced by N substrate, a few showed either no difference, or their differences were not well explained by N treatment clustering patterns. Thus, N availability likely has an impact on some, but not all, metabolic pathways in *M. aeruginosa* .

These findings show that the type of N substrate available affects *M. aeruginosa* 's cyanotoxin production dynamics, fitness strategies, and overall exometabolome composition. Results also have implications for understanding *M. aeruginosa's* interactions with other members of the cyanoHAB community and the progression of the bloom. N utilisation and cycling by *M. aeruginosa* and the associated microbial community are important for managing water bodies threatened by *Microcystis sp.‐dominated* cyanoHABs, particularly as fertiliser use shifts from primarily inorganic to organic N, and predicted climate changes may further favour *M. aeruginosa* over other phototrophs. In order to gain a more complete picture of the influence of N substrate on metabolism and ecological interactions in *M. aeruginosa*, further studies should build on these results and compare the effect of N on intracellular and extracellular metabolites, incorporate transcriptomics to track gene expression, and examine the relative effects of variables like N concentrations and light levels, which are likely to vary considerably in natural bloom communities. Additionally, because positive mode LC–MS favours N‐containing compounds or others likely to hold a positive charge, analysis using negative ion mode‐MS in future studies could be used to further expand our understanding of the metabolic response of *M. aeruginosa* to N source variation.

## Author Contributions

**Caroline M. Peck:** investigation, methodology, formal analysis, data curation, visualization, writing – review and editing, writing – original draft. **Lauren N. Hart:** methodology, formal analysis, visualization, writing – review and editing. **Roland Kersten:** resources, investigation, writing – review and editing. **Jenan J. Kharbush:** conceptualization, writing – original draft, writing – review and editing, resources, supervision, project administration, validation.

## Conflicts of Interest

The authors declare no conflicts of interest.

## Supporting information


**Data S1.** emi470189‐sup‐0001‐Supinfo.

**Data S2.** emi470189‐sup‐0002‐Tables.

## Data Availability

The data that support the findings of this study are openly available in MassIVE at https://doi.org/doi:10.25345/C5B27Q37C, MSV000096629. Additional data used in the study can be found in the Supporting Information. All code used to conduct analyses and data visualisation in the study can be found on GitHub at: https://github.com/cmpeck01/Peck‐et‐al.‐2025/tree/main.
