# Supplementary material for: Nitrogen Substrate Impacts Microcystis aeruginosa Exometabolome Composition
Source: Environ Microbiol Rep. 2025 Sep 2;17(5):e70189. doi: 10.1111/1758-2229.70189 (PMC12405062; doi:10.1111/1758-2229.70189)
Supplement: Supplementary file 1 — Data S1. emi470189‐sup‐0001‐Supinfo. [file EMI4-17-e70189-s001.docx]

**Supplementary Materials**

**SI Note 1:** *Protocol for making Liquid BG-11 2N or LE BG-11 2N for culturing Microcystis*

Notes on general preparation

- Only use acid-washed glassware when preparing stock and working solutions for culturing media.
- No precipitate should be present in stock solutions, dissolve completely using a magnetic stir bar.
- Once autoclaved, all solutions should **only** be opened in the laminar flow hood to ensure sterility.
- Check all stock solutions for contamination before use and remake as necessary.

Notes on medium

- BG-11 and BG-11 2N recipes were received from the Pasteur Culture Collection in Paris, France to culture *Microcystis sp.*
- The BG11-2N media is a variation on BG-11 where the nitrate concentration has been reduced to 2 mM and has been supplemented with 10 mM sodium bicarbonate to buffer the pH.

Media preparation: Prepare stock solutions (1/2)

NOTE: Prepare a 500 mL erlenmeyer flask, 125 mL erlenmeyer flask, 1 L pyrex bottle, and a magnetic stir bar.

1000x A5 Trace Metal Mix + Cobalt:

1. In a 500 mL erlenmeyer flask, dissolve 2.86 g boric acid (H_3_BO_3_) in 200 mL of milliQ water
2. Transfer to the 1 L glass bottle with magnetic stir bar.
3. Dissolve 1.81 g manganese (II) chloride tetrahydrate (MnCl_2_ ⋅ 4H_2_O) in 100 mL milliQ water
4. Transfer to the 1 L glass bottle while stirring.
5. Dissolve 222 mg zinc sulfate heptahydrate (ZnSO_4_ ⋅ 7H_2_O) in 100 mL milliQ water
6. Transfer to the 1 L glass bottle while stirring.
7. Dissolve 390 mg sodium molybdate dihydrate (Na_2_MoO_4_ ⋅ 2H_2_O) in 400 mL milliQ water
8. Transfer to the 1 L glass bottle while stirring.
9. In a 125 mL erlenmeyer flask
10. Dissolve 79 mg copper (II) sulfate pentahydrate (CuSO_4_ ⋅ 5H_2_O) in 50 mL milliQ water
11. Transfer to the 1 L glass bottle while stirring.
12. Dissolve 49 mg cobalt (II) nitrate hexahydrate (Co(NO_3_)_2_ ⋅ 6H_2_O) in 50 mL milliQ water
13. Transfer to the 1 L glass bottle while stirring.
14. Remove magnetic stir bar from 1 L glass bottle
15. Adjust volume to 1 L by adding 100 mL of milliQ water

Prepare stock solutions (2/2)

- 3.53 M sodium nitrate stock:
  - Dissolve 150 g solid sodium nitrate (NaNO_3_) in 500 mL milliQ water
- 200x potassium phosphate stock:
  - Dissolve 4 g potassium phosphate dibasic trihydrate (K_2_HPO_4_ ⋅ 3H_2_O) in 500 mL milliQ water
- 200x magnesium sulfate stock:
  - Dissolve 7.5 g magnesium sulfate heptahydrate (MgSO_4_ ⋅ 7H_2_O) in 500 mL milliQ water
- 200x calcium chloride stock:
  - Dissolve 3.6 g calcium chloride dihydrate (CaCl_2_ ⋅ 2H_2_O) in 500 mL milliQ water
- 200x Iron citrate stock:
  - Dissolve 300 mg of ferric ammonium citrate and 403 mg of citric acid trisodium salt anhydrous in 250 mL milliQ water. Protect this solution from light and replace monthly.
- 200x EDTA stock:
- Dissolve 100 mg of ethylenediaminetetraacetic acid disodium magnesium salt (K_2_ Mg EDTA) in 500 mL of water
- NOTE: at higher concentrations, EDTA will only dissolve in water at pH 8 or greater.
- 200x sodium carbonate stock:
  - Dissolve 2 g sodium carbonate (Na_2_CO_3_) in 250 mL milliQ water
- 1 M sodium bicarbonate stock:
  - Dissolve 42 g solid sodium bicarbonate (NaHCO_3_) in 500 mL milliQ water
  - Autoclave solution at 121℃ for 45 minutes.

Prepare working solution/ liquid media

- To a 1 L pyrex bottle add the following
- 500 mL milliQ or 0.2 μm filtered Lake Erie water
- 0.5 mL 3.53 M sodium nitrate stock
- 5 mL 200x potassium phosphate stock
- 5 mL 200x magnesium sulfate stock
- 5 mL 200x calcium chloride stock
- 5 mL 200x iron citrate stock
- 5 mL 200x EDTA stock
- 5 mL 200x sodium carbonate stock
- 1 mL 1000x A5 Trace Metal Mix + Cobalt

(NOTE: It is acceptable to have white precipitate/turbidity at this stage after adding sodium carbonate solution. Other kinds of precipitate suggest problems in media concentrations.)

- Adjust final volume to 1 L by adding 468.5 mL milliQ water
- Autoclave solution at 121℃ for 45 minutes.
- Allow the solution to cool to room temperature.
- In the biosafety hood, add 10 mL of sterile 1 M sodium bicarbonate stock to buffer pH.

(NOTE: This is done in the biosafety hood to ensure sterility.)

- Store at 4℃ in the dark for up to 3 months

**SI Figure 1:** Spectral matches for GNPS library hits. Top panel = this study, bottom panel = library spectrum.

**SI Figure 1.1: Microcystin LR**

 
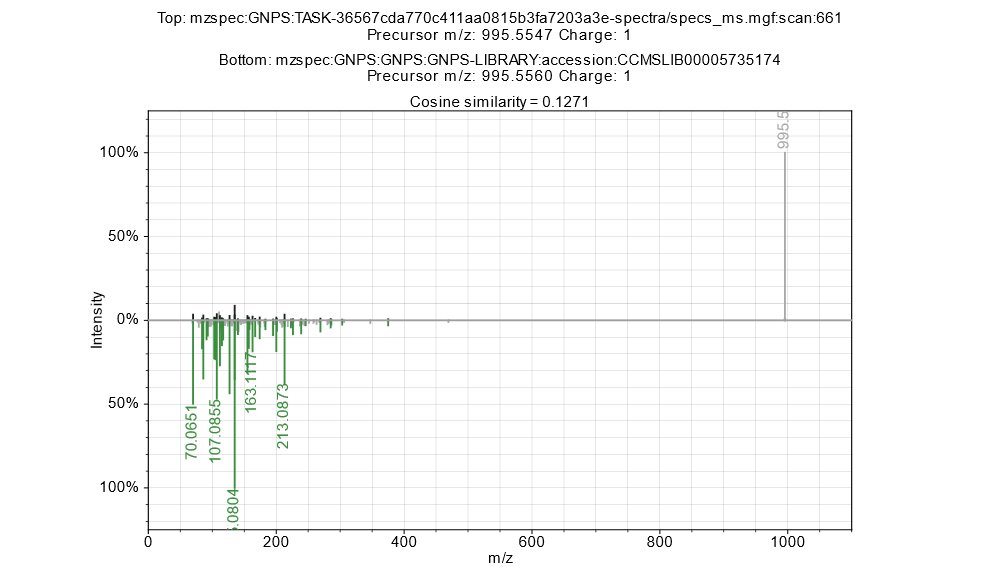


**SI Figure 1.2: Aerucyclamide A**

 
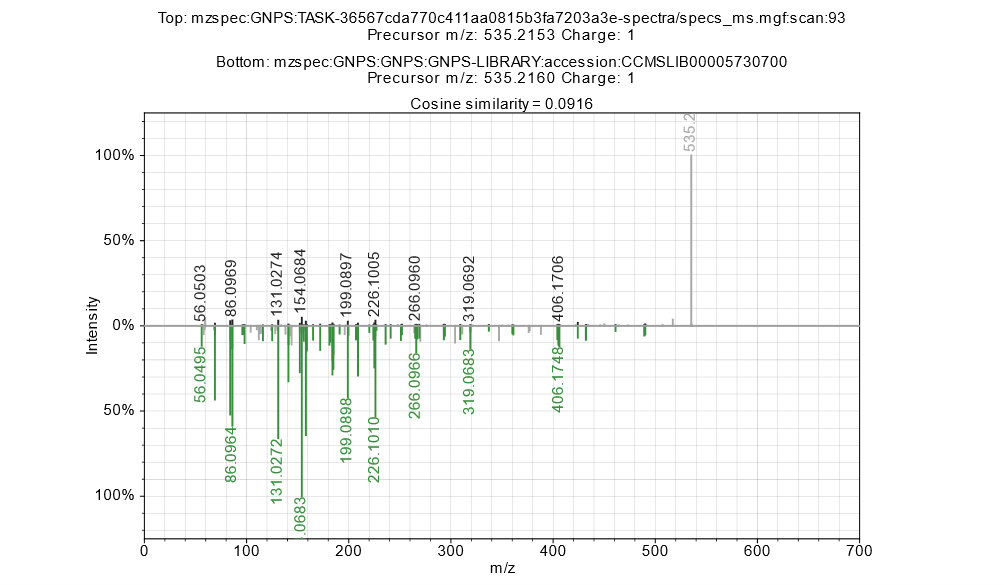


**SI Figure 1.3: Anabaenopeptin NZ857**

 
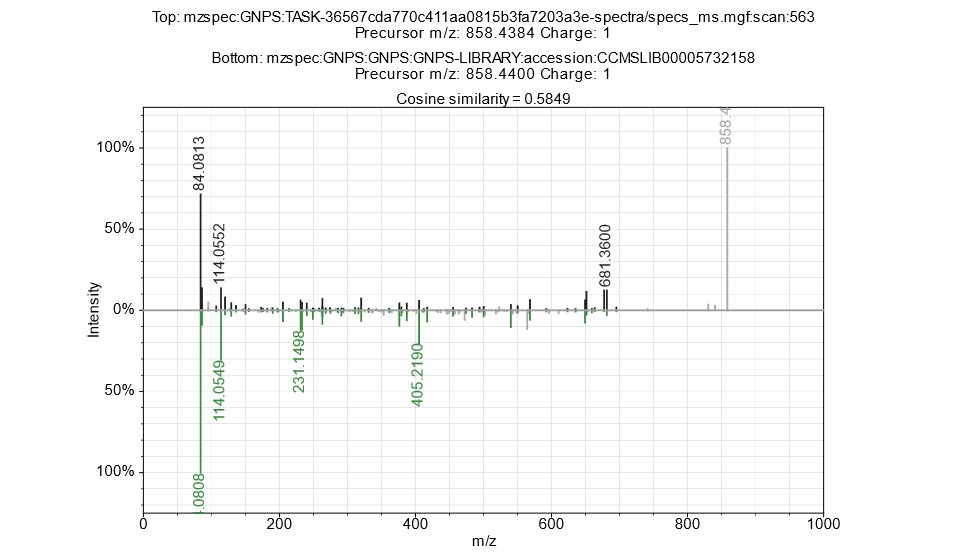


**SI Figure 1.4: Shinorine**

 
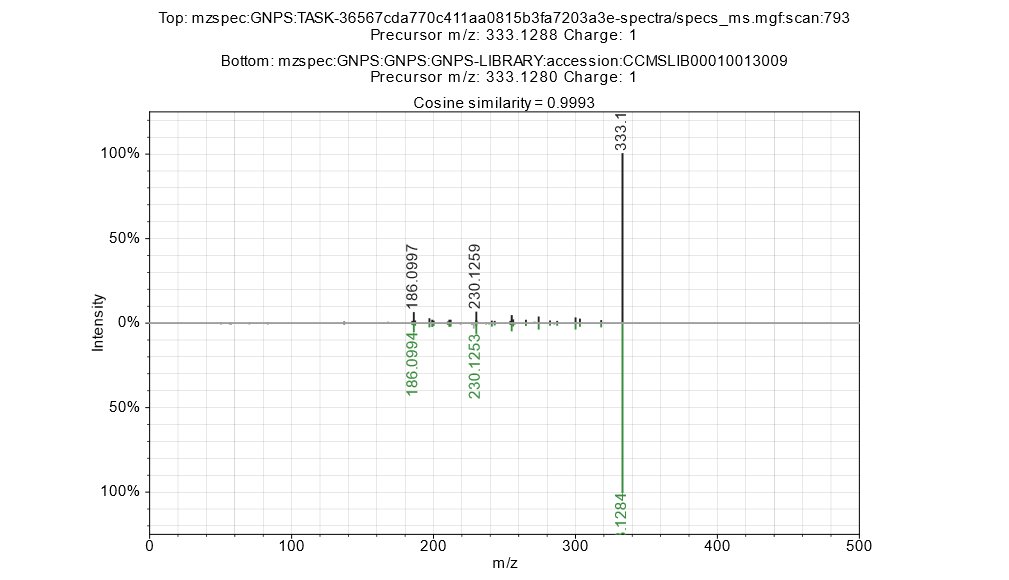


**SI Figure 1.5: Porphyra-334**

 
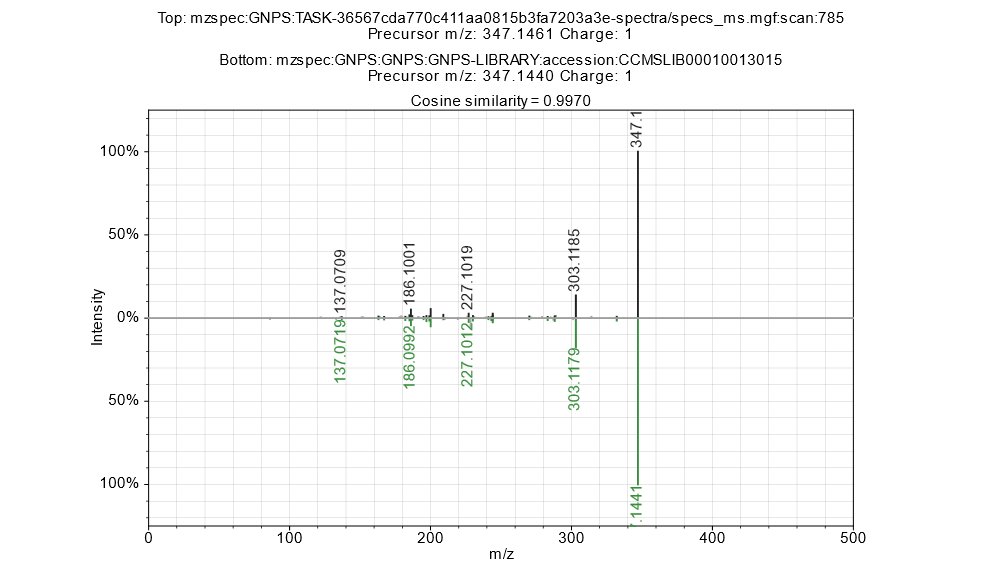


**SI Figure 1.6: β-Carotene**

 
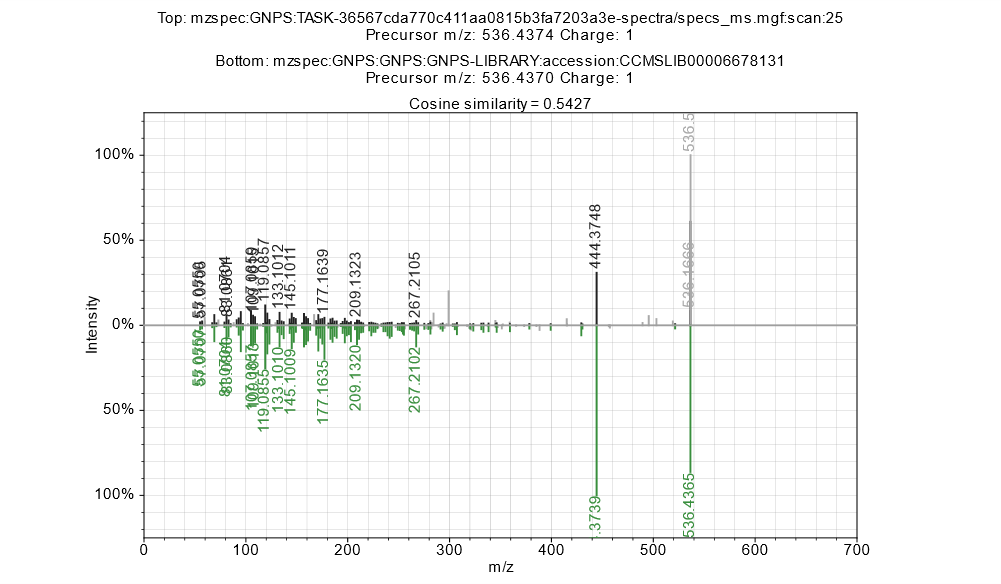


**SI Figure 1.7: Ergothioneine**

 
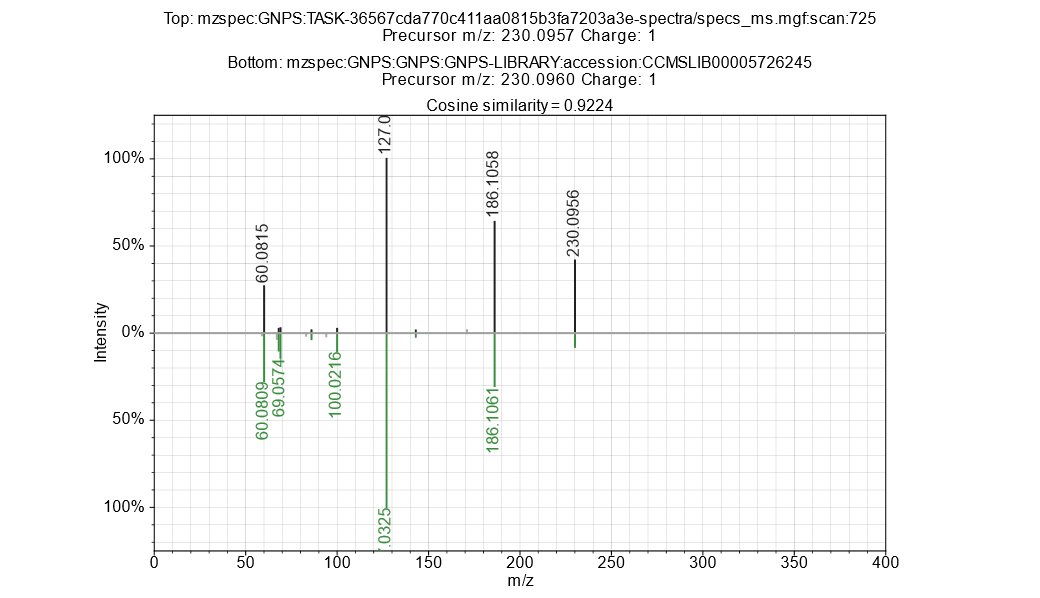


**SI Figure 1.8: 3-Deazauridine**

 
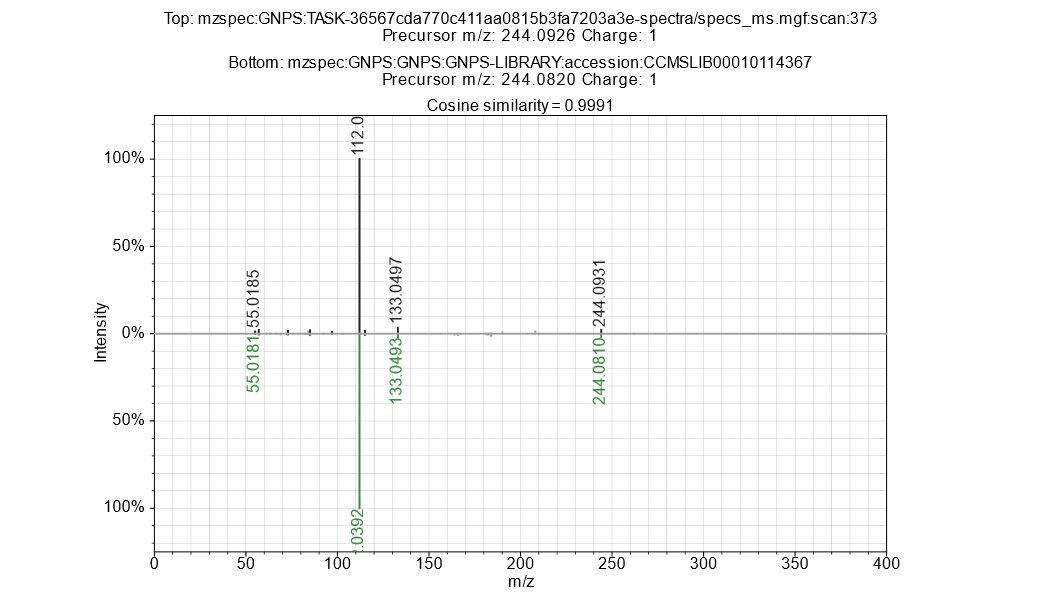


**SI Figure 1.9: 5’Methylthioadenosine**

 
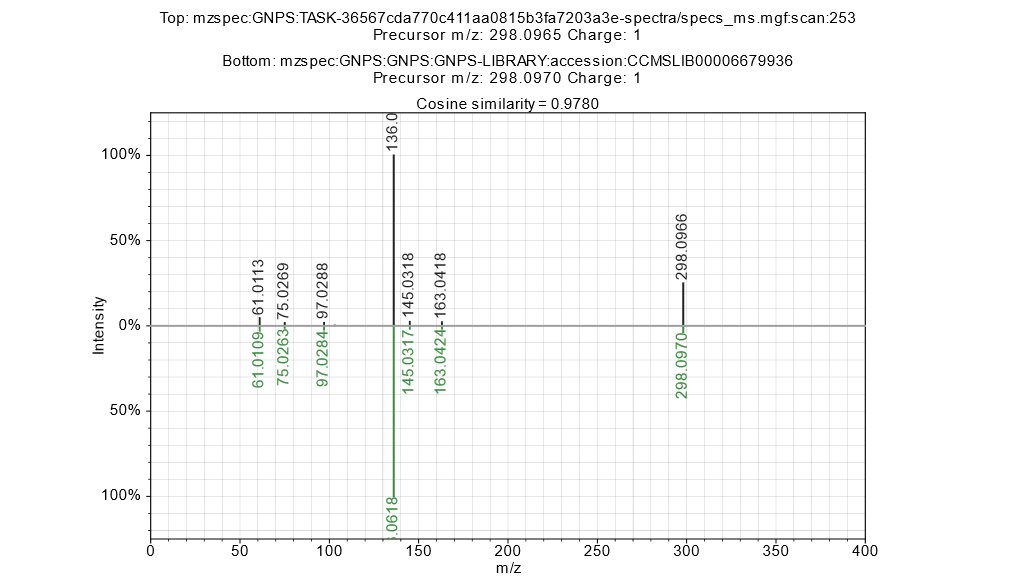


**SI Figure 1.10: L-Tryptophan**

 
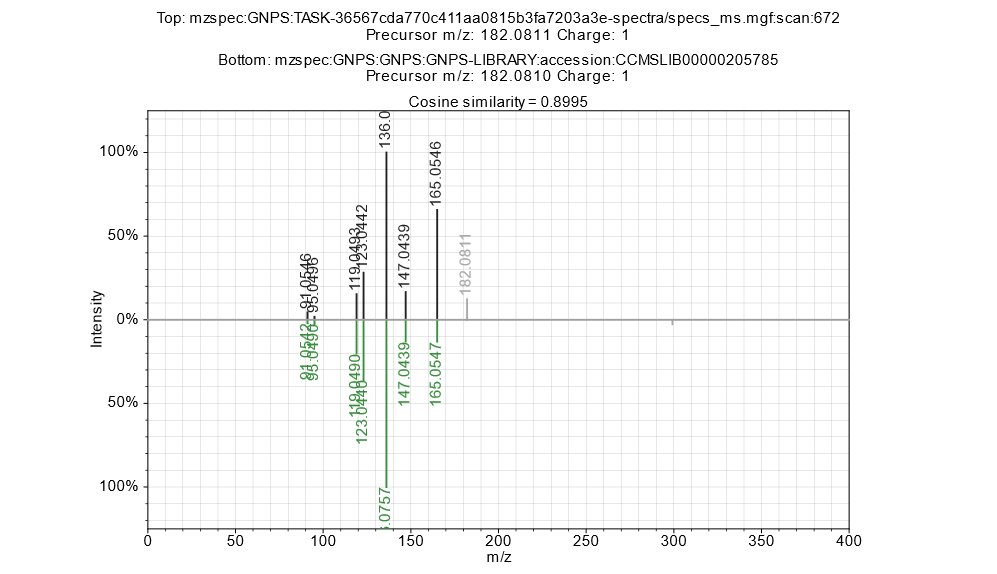


**SI Figure 1.11: Arginine**

 
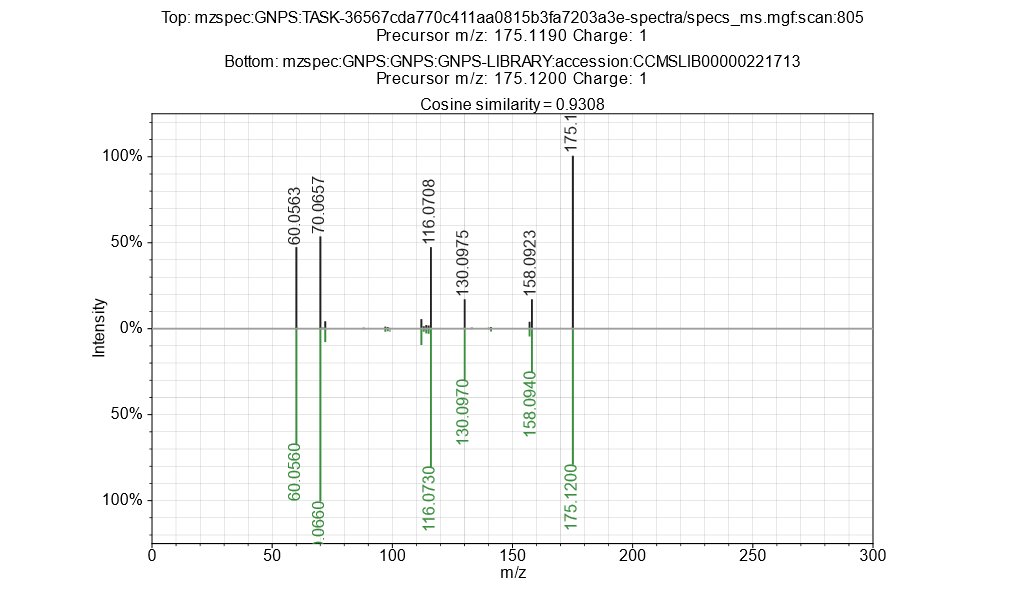


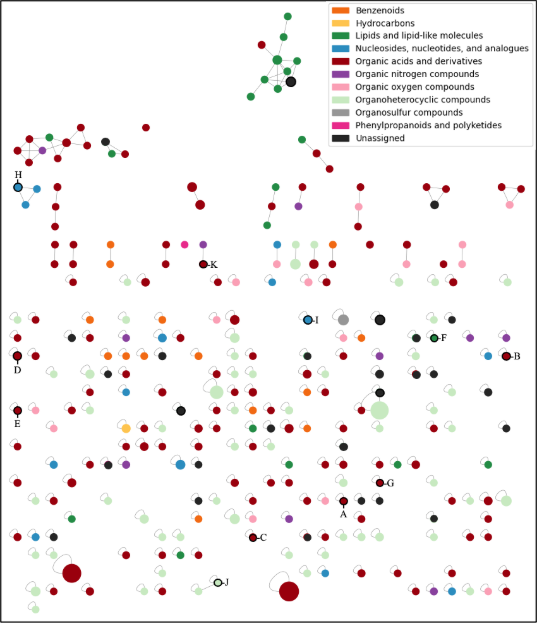


**SI Figure 2.** Molecular network of significant features across all experimental treatments. Each node represents a MS/MS spectrum, while each edge represents MS fragmentation similarity with cosine score greater than 0.7. Nodes are colored according to assigned ClassyFire SuperClass and scaled by relative peak area. Library hits are notated with letters.


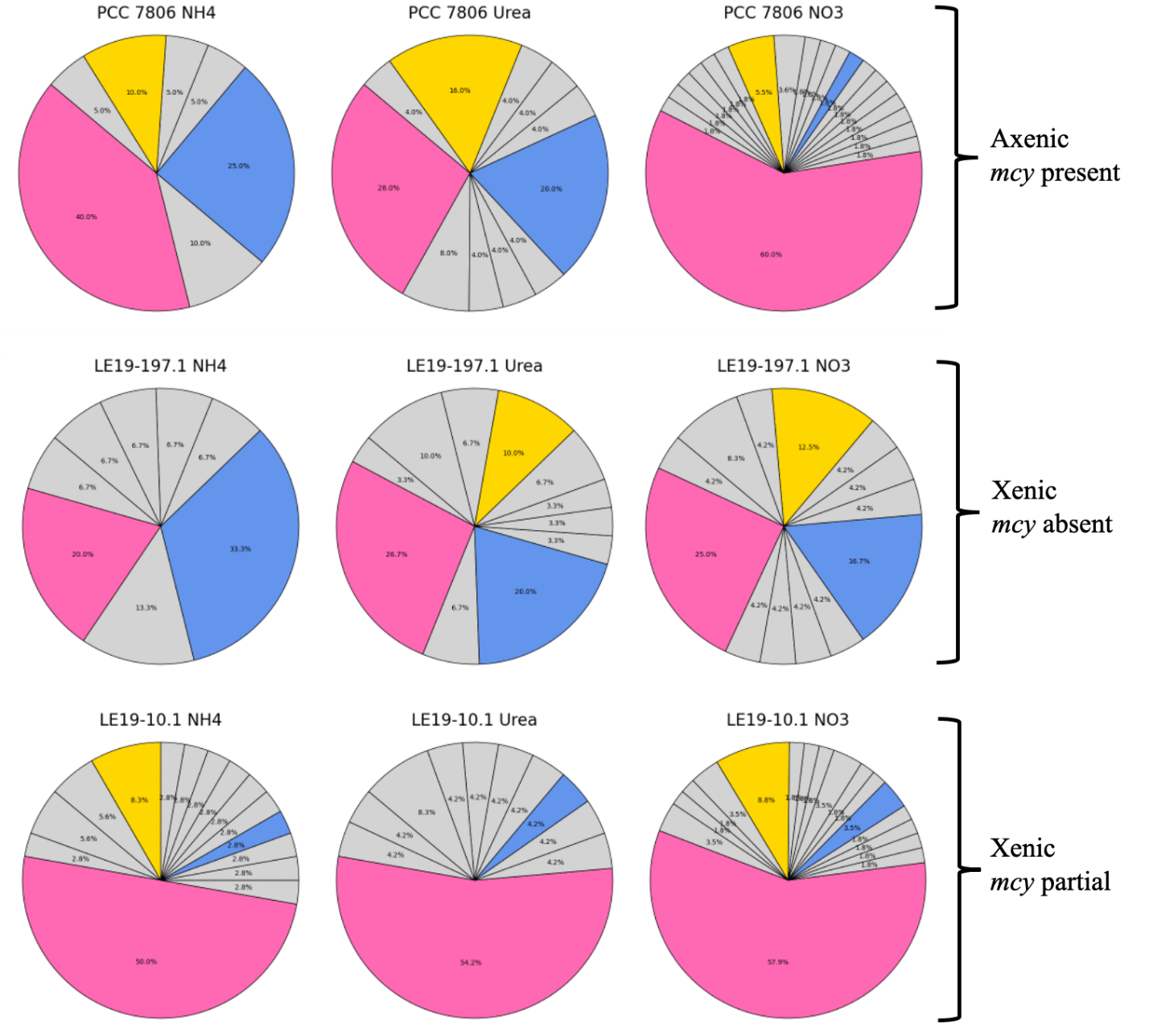


**SI Figure 3.** Comparison of SubClass composition percentage of only the metabolites produced in highly above average amounts across treatments. Compounds had center-scaled peak area values greater than 1. The three most represented SubClasses are colored; amino acids, peptides, and analogs in pink, purines and purine derivatives in yellow, and fatty acids and conjugates in blue.


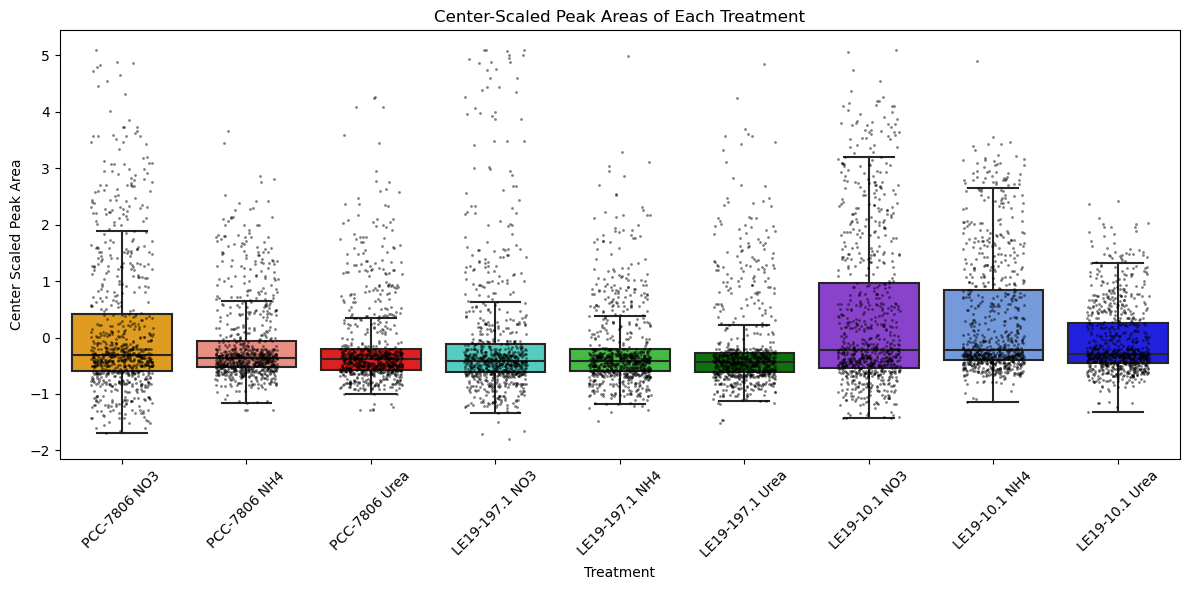


**SI Figure 4.** Determination of the cutoff for metabolites that were produced in “highly above average” amounts was made based on the highest Q3 threshold of all of the treatment groups.
